# Myositis facilitates preclinical accumulation of pathological prion protein in muscle

**DOI:** 10.1186/2051-5960-1-78

**Published:** 2013-12-03

**Authors:** Melanie Neumann, Susanne Krasemann, Katharina Schröck, Karin Steinbach, Markus Glatzel

**Affiliations:** Institute of Neuropathology, University Medical Center Hamburg-Eppendorf, Hamburg, 20246 Germany; Institute for Neuroimmunology and Clinical MS-Research (inims), Center for Molecular Neurobiology, Hamburg, Germany

## Abstract

**Background:**

In human and animal prion diseases, pathological prion protein, PrP^Sc^, as well as prion infectivity is mainly found in the central nervous system, but also in lymphoid organs and muscle. Pathophysiology of prion colonization of lymphoid organs has been studied intensively, yet how myositis influences prion accumulation in muscle is unknown.

**Result:**

We have investigated the influence of myositis on PrP^Sc^ accumulation and prion infectivity in two distinct mouse models of experimental autoimmune myositis. Furthermore, we have addressed the relevance of PrP^C^ expression in the lymphoreticular system in myositis by generating bone marrow chimeras.

Here we show that myositis positively influences muscular PrP^Sc^ accumulation at preclinical time points and that PrP^C^-expression in the lymphoid system is critical for this. In muscle, PrP^Sc^ and prion infectivity are uncoupled with detectable PrP^Sc^ but no prion infectivity at preclinical time points. Muscle has an intrinsically high ability to clear PrP^Sc^ once myositis has ceased, possibly involving autophagy.

**Conclusion:**

Our findings provide new insights into the pathophysiology of prion colonization in muscle pointing out that myositis leads to enhanced prion colonization of muscle in subclinical prion disease.

**Electronic supplementary material:**

The online version of this article (doi:10.1186/2051-5960-1-78) contains supplementary material, which is available to authorized users.

## Background

Prion diseases are characterized by the accumulation of misfolded prion protein (PrP^Sc^), a posttranslationally modified form of the host-encoded prion protein (PrP^C^) [1]. Accumulation of PrP^Sc^ correlates with neurodegeneration, and PrP^Sc^ represents an essential part of the infectious agent causing prion disease [2–4]. Prion diseases occur in animals as well as in humans and are transmissible within and more rarely between mammalian species. Prion diseases in animals include scrapie in sheep and goat [5], chronic wasting disease in elk and deer [6], and bovine spongiform encephalopathy in cattle [7]. Among the human prion diseases, three distinct etiologies are defined: they either arise sporadically as in sporadic Creutzfeldt-Jakob disease (sCJD), as autosomal dominantly inherited diseases as in genetic Creutzfeldt-Jakob disease (gCJD) or as acquired conditions in iatrogenic or variant Creutzfeldt-Jakob disease (iCJD, vCJD) [8, 9].

In prion diseases, accumulation of PrP^Sc^ and prion infectivity is not confined to the nervous system. PrP^Sc^ and prion infectivity are inevitably detectable in the lymphoreticular system (LRS) or in the skeletal muscle of terminally diseased individuals or animals [10–14]. In the subclinical phase of disease, the situation differs. Here PrP^Sc^ and prion infectivity are readily detectable in the LRS in the majority of instances, whereas prion accumulation in muscle occurs to a lesser extent both quantitatively and qualitatively [15–18].

Our knowledge on the pathophysiology of PrP^Sc^ accumulation between LRS and muscle differs as well. For prion colonization of the LRS, the molecular events have been worked out in great detail. Here, follicular dendritic cells (FDCs) residing in germinal centers of lymphoid follicles accumulate PrP^Sc^ before prions find their way to the central nervous system via peripheral nerves [19–21].

PrP^C^ expression in muscle has been studied and it could be shown that myocytes as well as muscle macrophages express PrP^C^[22] and that the expression in muscle is highly regulated and fibre-type specific [23]. Overexpression of PrP^C^ in muscle leads to a myopathy and a wide range of myopathies are characterized by increased PrP^C^-levels [23–27]. However, in muscle much less is known regarding molecular determinants of PrP^Sc^ accumulation. PrP^Sc^ accumulation is muscle-type specific with hind limbs showing higher PrP^Sc^ content than fore limb muscles [28]. Thus, the molecular mechanisms underlying accumulation of PrP^Sc^ and prion infectivity in skeletal muscle are poorly understood. Case studies from patients with myositis and prion disease suggest that inflammation may promote PrP^Sc^ accumulation [29]. In fact, recent data indicate that ectopic follicular inflammation is able to support prion accumulation even in non-lymphoid tissue [30]. On the other hand, peripheral nerves or muscle spindles or myocytes, have been shown to accumulate PrP^Sc^ even in the absence of inflammation [13, 15, 31].

Here, we show that PrP^Sc^ accumulation in skeletal muscle of mice was enhanced upon induction of experimental autoimmune myositis (EAM) in early subclinical prion disease. Our data suggest that accumulation of PrP^Sc^ correlates with elevated levels of PrP^C^ at the peak of myositis originating from infiltrating lymphocytes. Once myositis ceased, PrP^Sc^ was rapidly cleared from muscle most likely by autophagy which is upregulated in muscle compared to spleen and brain. Accumulation of PrP^Sc^ in inflamed muscle required presence of PrP^C^ on the LRS. Interestingly, titers of infectious prions as measured by bioassay were unchanged between the myositis and the control cohort pointing to an uncoupling of PrP^Sc^ loads and titers of infectious prions in our experimental model.

## Methods

### Animals

Five to six weeks old SJL/J and C57Bl/6 mice were purchased from Charles River Laboratory (Sulzfeld, Germany). Prion protein-deficient mice (*Prnp*^*0*/*0*^) [32] were bred in house. Mice were sacrificed in groups of 4 animals at day 35, day 60, and day 90 after inoculation with Rocky Mountain Laboratory (RML) prions or when clinical signs of terminal prion disease (tail rigidity, weight loss, ataxia, and roughened fur) occurred. All animal procedures were performed in accordance with the institutional guidelines from the animal facility of the University Medical Center Hamburg-Eppendorf.

### Generation of bone marrow chimeras

C57Bl/6 mice were irradiated with a dosage of 8 Gy with constant 1 Gy/min using a BIOBEAM 2000 Cs-137 chloride gamma irradiator (Eckert & Ziegler, Berlin, Germany) one day prior to bone marrow reconstitution. Bone marrow was harvested in serum-free media (D-MEM) at 4°C from femur and tibia of the hind-legs of *Prnp*^*0*/*0*^ and C57Bl/6 mice using a syringe with a 23 G needle. The cell suspension was transferred to a 100 μm strainer and the flow-through was centrifuged at 300 × g for 10 min at 4°C. Cell pellets were solubilised in 1 ml of erythrocytes-lysis-buffer containing 0.15 M NH_4_Cl, 10 mM KHCO_3_, and 0.1 mM Na_2_EDTA (pH = 7.2 to 7.4) and were then incubated for 5 min on ice. Afterwards cells were washed with PBS and transferred to a 40 μm strainer. The flow-through was directly used for reconstitution. Each mouse was injected intravenously with 300 μl of the cell suspension containing about 10^7^ bone marrow cells in total and reconstitution efficiency was assessed by FACS analysis (see below).

### Induction of an experimental autoimmune myositis (EAM)

In order to induce an EAM, SJL/J mice were treated as described before [33]. Briefly, purified myosin from rabbit skeletal muscle (6.6 mg/ml; Sigma-Aldrich, Munich, Germany) was emulsified with an equal amount of Complete Freund's adjuvant (CFA) (Difco Laboratories, Detroit, USA) with 3.3 mg/ml *Mycobacterium butyricum* (Difco Laboratories, Detroit, USA). Mice were anesthetized using a CO_2_/O_2_ mixture and were then immunized subcutaneously with 100 μl of the emulsion into four locations (total, 400 μl) on the back on days 0, 7, and 14. On day 21 and 54 mice were either sacrificed for histological and FACS analysis or they were inoculated at day 21 with RML prions. In order to introduce an EAM in C57Bl/6 mice, in addition animals were injected intraperitoneally with 0.5 μg Pertussis toxin in PBS at all time points of immunizations.

### Inoculations, determination of incubation time and prion titers

*At d*ay 21 mice were anesthetized using an intraperitoneal injection of ketamine (12 mg/ml) mixed with xylazine (1.6 mg/ml) with a dosage of 100 μl solution per 10 g bodyweight. Afterwards mice were inoculated intraperitoneally with 100 μl PBS containing 6 logLD_50_ units of RML scrapie strain. In order to determine the incubation time to onset of terminal prion disease, mice were kept until the day of onset of terminal clinical signs of prion disease (see above). Incubation time in days starting from the day of scrapie administration until the day of death was determined and plotted against the survival probability in % in Kaplan-Meier survival curves using SPSS statistic software.

Bioassays to determine titers of prion infectivity were performed on 1% homogenates of either spleen or muscle tissue. Spleens from one single animal per group and muscle tissues from either 1 animal per group (muscle control samples but day 90 and all chimeras), 2 animals per group (SJL/J mice day 35, day 60 and terminal diseased) or 3 animals per group (SJLJ/ mice day 90) were homogenized in 0.32 M sucrose using a FastPrep FP120 homogenizer (Qbiogene, Cedex, France), diluted in 5% BSA in PBS and centrifuged for 5 min at 500 × g. 30 μl of each supernatant were inoculated intracerebrally into groups of 4 highly prion susceptible tga20 mice [34]. The relationship y = 11.45-0.088*x (y, log LD_50_ per milliliter of homogenate; x, incubation time in days to terminal disease) was used to calculate prion titers [35, 36].

### Immunohistochemistry

**Figure 1:**
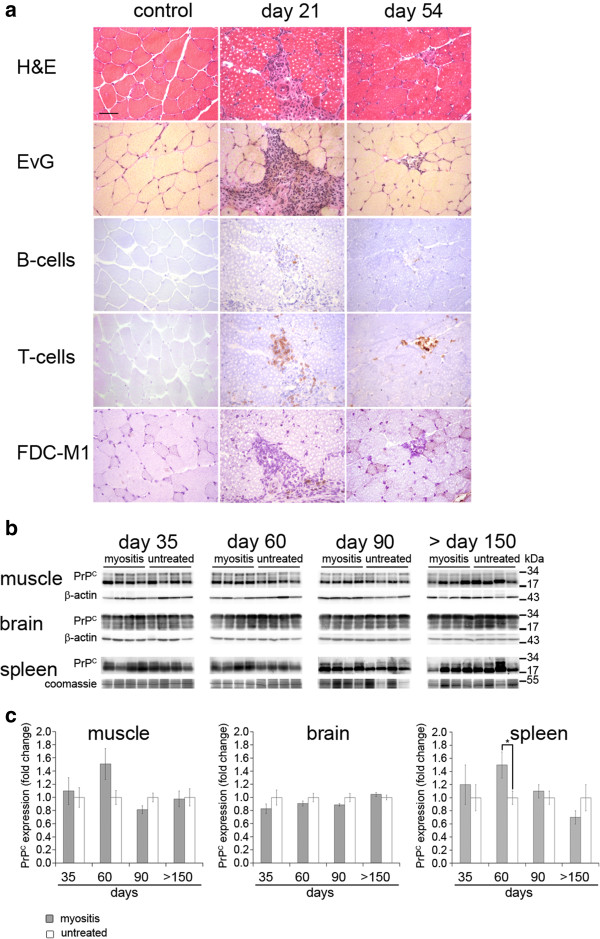
**Analysis of muscle tissue derived from myositis-induced SJL/J mice and untreated controls. a)** H&E- as well as Elastica-von-Giesson staining show a high number of infiltrating immune-cells at day 21 after the immunization with myosin, which is followed by a milder invasion of inflammatory cells and necrosis of single muscle fiber at day 54. Immunohistochemical stainings of B-, T-cells and FDCs reveal that most of the infiltrating cells are T-cells and that only very few B- cells or FDCs can be detected. (Scale bar is 50 μm). **b)** Western blot analysis of PrP^C^ of muscle, brain and spleen. Beta-actin (for muscle and brain) and coomassie-staining (for spleen) are shown as loading controls (molecular weights indicated in kDa). **c)** Quantification of PrP^C^ expression in muscle, brain and spleen. Values were adjusted to loading controls. Note that there is a peak of PrP^C^ expression at day 60 in muscle as well as in spleen in myositis-induced mice.

**Figure 2:**
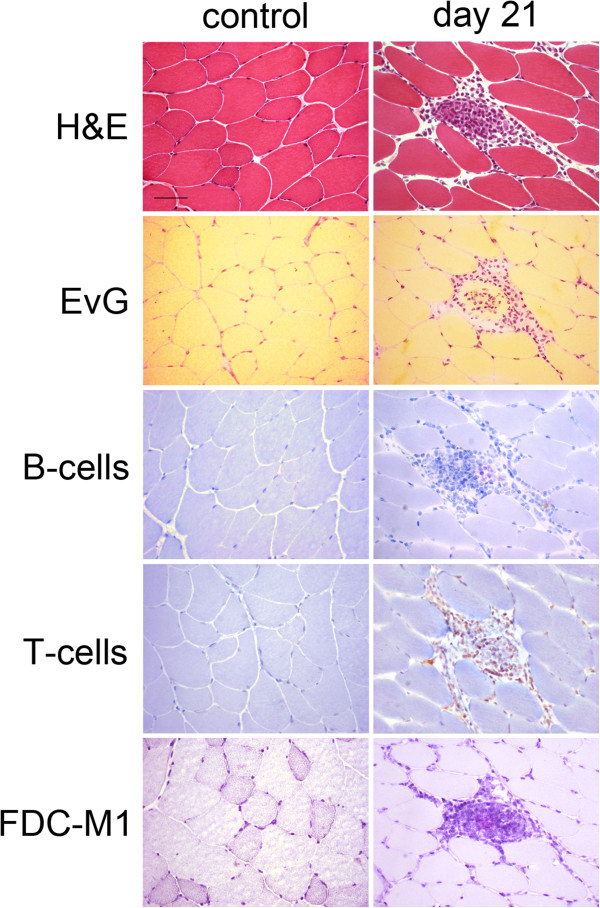
**Analysis of muscle derived from C57Bl/6 mice with EAM and controls.** H&E- as well as Elastica-von-Giesson staining show a mild infiltration of immune-cells at day 21 after the immunization with myosin. Immunohistochemical stainings of B-, T-cells and FDCs reveal that most of the infiltrating cells are T-cells and that only very few B-cells and no FDCs can be detected. (Scale bar is 50 μm).

**Figure 3:**
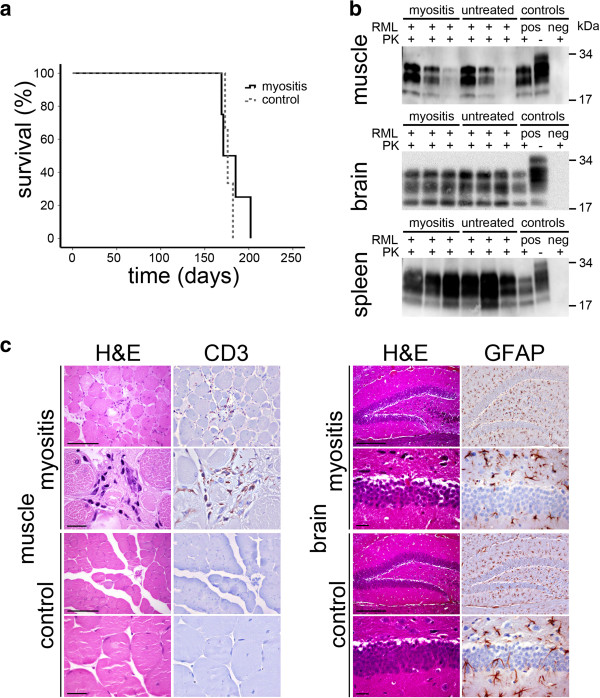
**Analysis of terminal diseased EAM / control mice. a)** Kaplan-Meier curve showing no differences in survival probability in prion inoculated EAM-mice when compared to controls. **b)** Western blots of muscle, brain and spleen of terminal sick EAM / control mice. PrP^Sc^ loads are variable without significant differences between groups. As positive control, brain homogenate from a terminally diseased wild type mouse is loaded with and without PK-digestion. As negative control brain homogenate from a healthy wild type mouse is loaded with PK-digestion (molecular weights indicated in kDa). **c)** H&E as well as immunohistochemical staining for T-cell marker CD3 show signs of myositis in the muscle tissue only in EAM mice but not controls. Nevertheless, in brain tissue in both cohorts a pronounced gliosis could be detected by H&E and immunohistochemical staining for astrocyte marker GFAP (Scale bar is 100 μm and 20 μm for muscle and 200 μm and 20 μm for brain).

**Figure 4:**
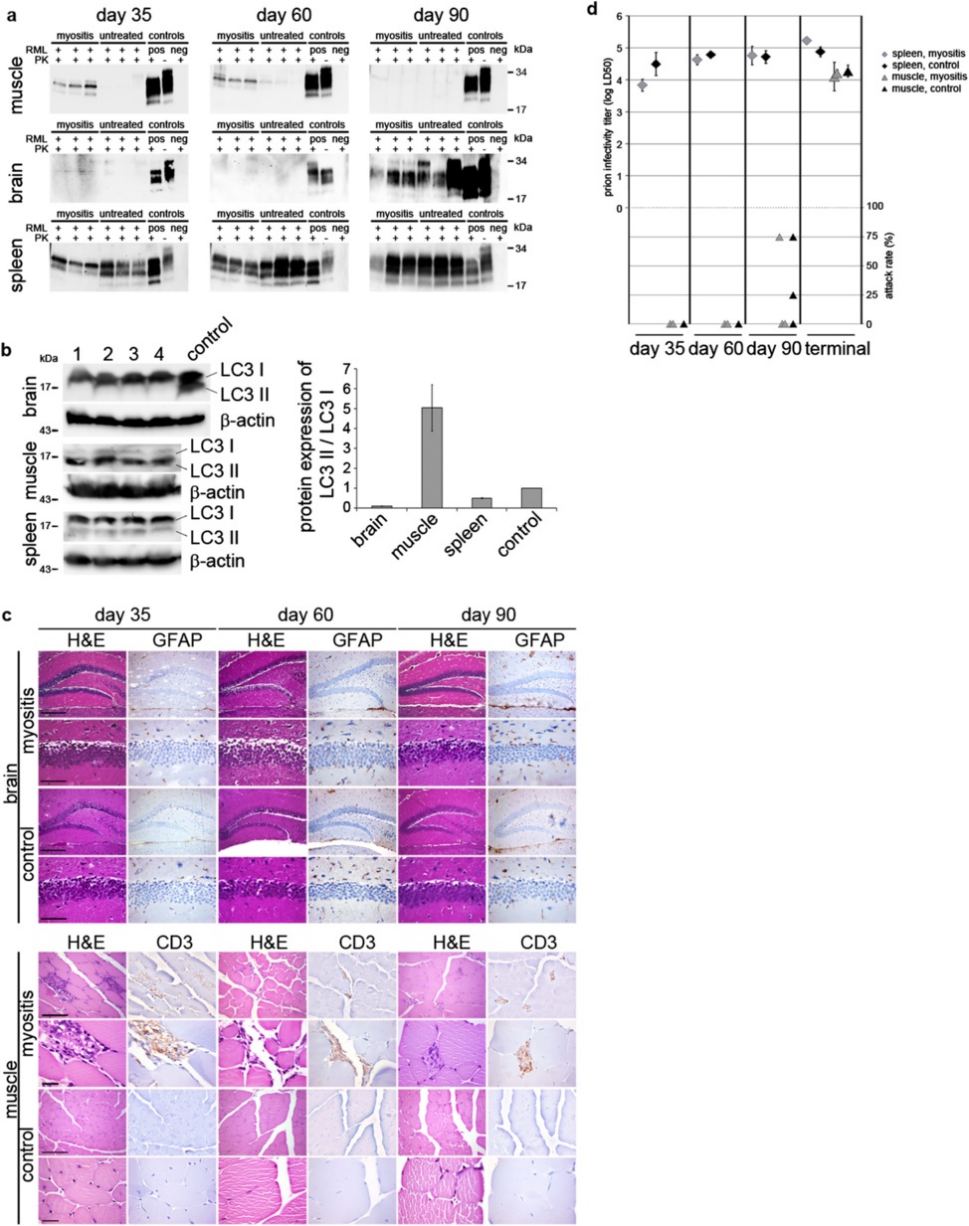
**Analysis of preclinical prion-inoculated EAM / control mice. a)** NaPTA blots (muscle and brain) as well as regular Western blots (spleen) show PrP^Sc^ load over time in EAM versus untreated mice. As positive controls for NaPTA blots RML is used as a spike in untreated tissue homogenate and is either PK-digested or undigested. For blot of spleen brain homogenate from a terminally diseased wild type mouse is loaded with and without PK- digestion. As a negative control, PK-digested normal tissue homogenate is used. Note that there are differences in PrP^Sc^ loads in muscle at day 35 and 60 in EAM versus control mice. **b)** Quantifications of western blots of autophagy marker LC3 show elevated amounts of LC3 II versus LC3 I in muscle versus spleen and brain of C57/Bl6 mice at day 0. (n = 4; control = brain homogenate of a cathepsin D knockout mouse). **c)** H&E as well as immunohistochemical staining for CD3 show signs of myositis in the muscle tissue only in treated mice in contrast to untreated controls. In brain tissue in both cohorts no gross pathological hallmarks of prion disease could be detected by H&E and immunohistochemical staining for GFAP over time (scale bar is 200 μm and 20 μm for brain and 100 μm and 20 μm for muscle). **d)** Bioassays to determine titers of prion infectivity reveal no differences between both prion-inoculated EAM and prion-inoculated control mice.

**Figure 5:**
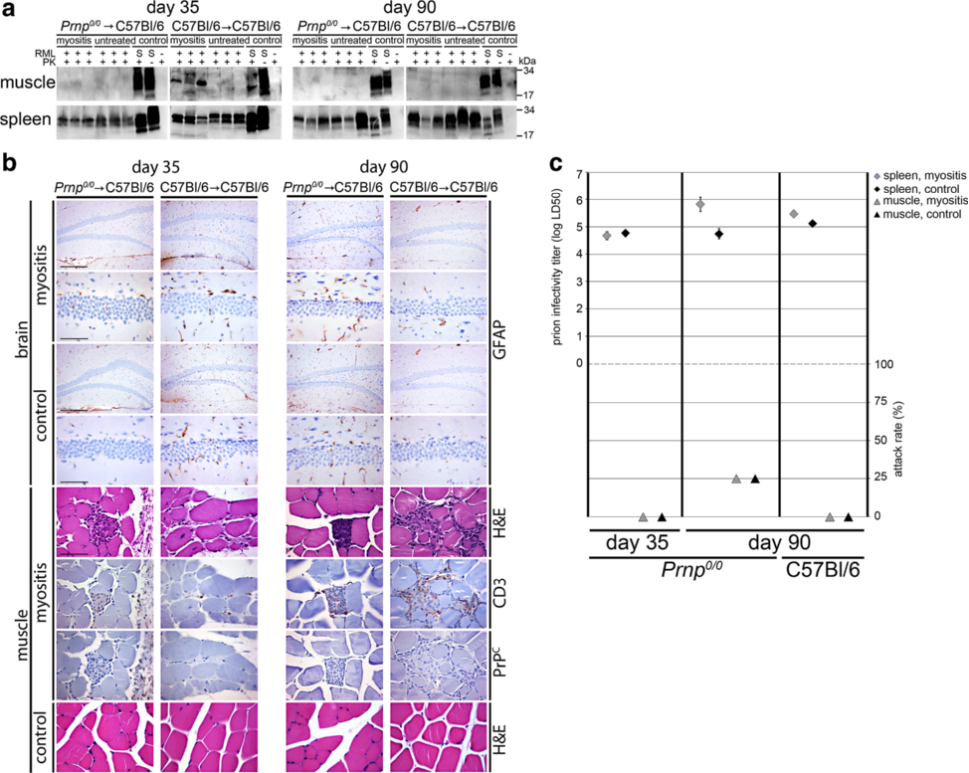
**Analysis of preclinical prion-inoculated EAM / control mice with either Prnp** ^**0/0**^ **or wt LRS. a)** NaPTA blots of muscle and Western blots of spleen for PrP^Sc^ over time in EAM versus control mice. As positive controls for NaPTA blots, RML is used as a spike (S) in untreated tissue homogenate and is either PK-digested or undigested. As positive controls for spleen, brain homogenate from a terminally diseased wild type mouse is loaded with and without PK-digestion. As a negative control, PK-digested normal tissue homogenate is used. Muscular PrP^Sc^ can only be found in EAM mice with PrP^C^ expressing LRS at day 35. At day 90, no PrP^Sc^ could be detected in both EAM and control mice. In spleen PrP^Sc^ is detectable in all analyzed samples. **b)** Histological staining with H&E evidences signs of myositis in the muscle of both chimeric mice with EAM but not controls. The staining for marker CD3 shows that most of the infiltrating cells are T-cells. Anti-PrP^C^ staining could not be detected most probably because its expression is under the detection limit of the method. In brain tissue in both cohorts no gross pathological hallmarks of prion disease like spongiosis or gliosis could be detected by immunohistochemical staining for astrocyte marker GFAP. **c)** Bioassay to determine prion infectivity titers reveal high infectivity titers for all analyzed spleen samples. In muscle, prion titers were under the detection limit of the assay at day 35 irrespective of PrP^C^ expression in the LRS. At day 90 borderline infectivity (attack rate of 25%) could be detected in mice with PrP^C^-deficient LRS irrespective of myositis.

Muscle and brain tissues were either frozen in liquid nitrogen-cooled 2-methyl-butan (Sigma Aldrich, Munich, Germany) and cut into 8 μm sections with a cryostat (CM1950, Leica, Wetzlar, Germany; see Figures 1a and 2), or were fixed using 3.5% of formaldehyde solution buffered according to Lillie for 15 hrs (see Figures 3c, 4c and 5b). If appropriate, tissues were prion-inactivated using 98% formic acid for 1 hr followed by 3.5% of formaldehyde solution for at least 12 hrs. Tissues were processed to paraffin blocks using an ASP300S dehydration machine (Leica, Wetzlar, Germany) and an EG1160 tissue embedding system (Leica, Wetzlar, Germany). Paraffin blocks were cut into 4 μm sections, which were stained with hematoxylin and eosin following standard laboratory procedures. Sections from frozen tissue were also stained with Elastica-von-Giesson standard staining solutions. For immunohistochemical staining the Ventana Benchmark XT machine (Ventana, Tuscon, Arizona, USA) was used. Briefly, deparaffinised sections were boiled for 30 to 60 min in 10 mM citrate buffer, pH 6.0, for antigen retrieval. Primary antibodies were diluted in 5% goat serum (Dianova Immundiagnostic, Hamburg, Germany), 45% Tris buffered saline pH 7.6 (TBS) and 0.1% Triton X-100 in antibody diluent solution (Zytomed, Berlin, Germany). Sections were then incubated with primary antibody for 1 hr (see also Table 1). Anti-rabbit or anti-goat histofine Simple Stain MAX PO Universal immunoperoxidase polymer (Nichirei Biosciences, Wedel, Germany) were used as secondary antibodies. Detection of secondary antibodies and counter staining was performed with an ultraview universal DAB detection kit from Ventana (Ventana, Tuscon, Arizona, USA) according to the standard settings of the machine. The staining of FDCs was performed according manufactures’ instructions. All sections were cover-slipped using TissueTek® glove mounting media (Sakura Finetek, Staufen, Germany), and dried in an incubator at 60°C. Pictures were taken using a light microscope (Axioskop 40, Zeiss, Jena, Germany or Olympus BH-2, Hamburg, Germany) equipped with a digital camera (AxioCam ICc3 Zeiss, Jena Germany).

**Table 1:** **List of primary antibodies**

Name	Specificity	Dilution	Company
B220	B-cells	1:400	eBioscience
CD3	T-cells	1:100	Thermo Fisher Scientific
GFAP	astrocytes	1:200	Dako
MFG-E8	follicular dendritic cells	1:50	BD Bioscience
LC3	autophagy	1:200	nanoTools
POM-1	PrP^C^ and PrP^Sc^	1:1000	kindly provided by A. Aguzzi
beta-Actin	cytoskeleton	1:1000	Millipore

### Sodium phosphotungstic acid (NaPTA) precipitation

NaPTA precipitation was performed according to a previously, slightly modified method [14, 37]. Briefly, 100 mg of frozen tissue was thawed and put into 900 μl of dissociation buffer containing 25 mM HEPES (pH 7.2), 0.3 M sucrose and 53.6 μg Liberase Blendzyme 2 (Roche, Penzberg, Germany). Samples were incubated for 30 min at 37°C with a ribolyzing step performed every 10 min, until completely homogenized. To each 500 μl of 10% (w/v) tissue homogenates an equal volume of 4% (w/v) sarkosyl-PBS was added, vortexed, and incubated for 10 min at 37°C with constant agitation. 50 U/ml of benzonase (Novagen, Darmstadt, Germany) and 1 mM MgCl_2_ were added, and incubated at 37°C for 30 min with vigorous agitation. Afterwards, 81.3 μl of a pre-warmed (to 37°C) 4% (w/v) NaPTA/170 mM MgCl_2_ solution (pH 7.4) was added, vortexed, and incubated with vigorous agitation for 30 min at 37°C. Samples were then centrifuged at 16,000 × g for 30 min, after which supernatants were carefully removed and the remaining pellets resuspended in 22.5 μl of 0.1% sarkosyl-PBS. For detection of PrP^Sc^ only, samples were digested with proteinase K (Invitrogen, Karlsruhe, Germany) for 60 min at 37°C with a final concentration of 20 μg/ml. Since PrP^C^ is proteinase K sensitive it is digested completely after this treatment, whereas PrP^Sc^ is proteinase K resistant and therefore is still detectable. Digestion was stopped by adding CVL-sample buffer (1% (w/v) SDS; 25 mM Tris/HCl, pH 7.4; 2,5% (v/v) β-mercaptoethanol; 1.5% (w/v) sucrose; 0.02% (w/v) brome-phenol-blue) and boiling for 10 min prior to Western blot analysis. As positive control RML 5.0 standard inoculum brain homogenate (RML) was spiked into uninfected tissue and digested as described.

### Western blots

Frozen tissues were thawed, homogenized and ~50 μg of total protein were separated by SDS-PAGE on a 12% SDS-PAGE gel, transferred to PVDF membranes (Biorad, Munich, Germany) at 400 mA for 1 hr in a wet-blot chamber (Biorad, Munich, Germany), and blocked for 1 hr at room temperature in 5% (w/v) milk powder in PBS containing 0.1% of Tween 20. Membranes were incubated overnight at 4°C using anti-PrP antibody POM1 or for two days using the anti-LC3 antibody, both in blocking buffer (see also Table 1). After incubation for 1 hour at room temperature with an HRP-conjugated anti-mouse secondary antibody (Promega GmbH, Mannheim, Germany, 1:5000 in blocking buffer), proteins were detected using ECL femto reagent (Pierce, Rockford USA). Samples were recorded and chemiluminescence signals were quantified using a chemiluminescence reader (Biorad, Munich, Germany). Detection of beta-actin (Sigma-Aldrich, Seelze, Germany) served as an internal loading control.

### FACS analysis of blood samples

Two drops of blood, drawn by submandibular puncture were transferred into 5 ml of FACS buffer containing 2% (v/v) FCS, 0.01 M EDTA (pH 8) and 0.1% (w/v) NaN_3_ in PBS. After centrifugation of the samples for 10 min at 1000 rpm supernatants were discharged and 100 μl of primary antibody (biotinylated 6H4, Prionics, Planegg-Martinsried, Germany) diluted 1:100 in FACS buffer was added to the pellets and incubated at 4°C for 30 min. Afterwards samples were washed and incubated with fluorochrome-labelled streptavidin diluted 1:100 in FACS buffer at 4°C for 30 min. After another washing step samples were counterstained with an antibody against T-cells (PE-anti-CD3 (2 μl per sample)) by incubating at 4°C for 30 min. Following rigorous washing steps, samples were resuspended in 0.5 ml of FACS buffer and were analyzed using a LSRII FACS machine (BD Bioscience, Heidelberg, Germany).

### FACS analysis of muscle samples

Quadriceps femoris muscles of immunized mice were dissected, minced and put into 5 ml of digestion buffer before incubation at 37°C for 30 min. Then, cooled (4°C) samples were washed and passed through a 100 μm cell strainer as well as through a 30 μm pre-separation filter (Miltenyi Biotec, Bergisch Gladbach, Germany). The flow-through was resuspended in 2.5 ml of a 30% Percoll solution and underlayed with 2 ml of a 78% Percoll solution before centrifugation for 30 min at 2500 rpm. Cells lying at the interface of the gradient were removed and washed several times with FACS buffer. In order to block Fc receptors before FACS analysis, pellets were incubated for 10 min in 50 μl of FACS buffer containing 1 μl of Fc-block (eBioscience, Frankfurt, Germany). After washing cells were stained for 1 hr using 10 μl of each of the following antibodies: Gr1-FITC, NU1.1-PE, B220-PECP-Cy5.5, CD11c-PECy7, CD11b-APC, CD45-APCCy7, and CD3-PaBkuc. After washing cells were resuspended in 400 μl of FACS buffer and were analyzed using a LSRII FACS machine (BD Bioscience, Heidelberg, Germany).

### Statistical analysis

In all experiments, mean +/− SEM is reported. Statistical comparisons among groups were determined using Student’s *t* test.

## Results

### Induction of experimental autoimmune myositis (EAM) in SJL/J mice

**Figure 6:**
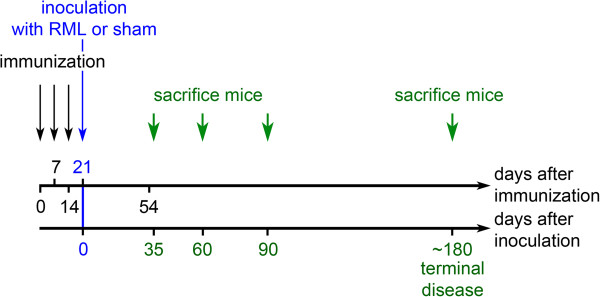
**Schedule of the experiments.** The upper timeline shows the days after immunization of the mice, whereas the lower timeline displays the time after inoculation with RML or sham. Note that the day of inoculation is set to 0 again in order to calculate the exact days until terminal disease.

EAM was induced by subcutaneous injection of myosin and CFA at days 0, 7 and 14. On day 21 and 54, quadriceps femoris muscles of hind-legs were analyzed by FACS or histology (Figure 6). As shown in Table 2, FACS revealed T-cell dominated myositis (50.3 and 21.3% of all infiltrating cells at day 21 and 54 were CD3-positive; 17.3 and 1.4% of all infiltrating cells at day 21 and 54 were B220-positive) in EAM mice when compared to controls (5.6% of all infiltrating cells were CD3-positive; 1.2% of all infiltrating cells were B220-positive). Neutrophil granulocytes and other lymphocytes were not changed between EAM and controls. Up-regulation of T- and B-cells in EAM could be confirmed histologically (Figure 1a), where we found prominent lymphocytic infiltrates as early as day 21. Immunohistochemical staining with antibodies against CD3 for T-cells, B220 for B-cells and milk fat globule protein-epidermal growth factor-8 (MFG-E8) for FDCs demonstrated high numbers of T- and B-cells as well occasional MFG-E8-positive cells in EAM when compared to control tissue. Less pronounced infiltrates of T- and B-cells, but no MFG-E8-positive cells could be detected at day 54 after immunization. At both time points, morphological signs of chronic myositis such as necrotic muscle fibers and fibrosis were found in EAM but not in controls (Figure 1a). Interestingly, myositis did not lead to a reduction in muscle strength as demonstrated by hanging wire test (Additional file 1: Figure S1). For further analysis, prion inoculations were performed at peak of inflammation (21 days after the first immunization). For reasons of clarity, day of RML/sham (CD1 brain homogenate) inoculation was set to day 0, see Figure 6.

**Table 2:** **FACS analysis of muscle homogenates from SJL**/**J mice with EAE** / **control at day 21 and 54 after induction of EAE**

Infiltrating cells (in %)	Control	21 days	54 days
T-cells	5.6	50.3	21.3
B-cells	1.2	17.3	1.4
Other lymphocytes	5.0	6.0	3.0
Neutrophile granulocytes	18.9	9.4	19.8

Values are the percentages of every immune cell regarding to the total amount of infiltrating cells.

### EAM leads to enhanced expression of PrP^C^ in muscle

To investigate if EAM leads to an up-regulation of PrP^C^ in muscle, we analyzed muscle tissue for its PrP^C^ content by Western blotting and compared data to PrP^C^-levels in spleen and brain. As shown in Figure 1, we could detect a slight up-regulation of PrP^C^ at day 60 after sham immunization in muscle (Figure 1b,c; p = 0.1). In all other muscle samples we could not find any changes in PrP^C^ expression. As expected, brain showed high levels of PrP^C^ irrespective of EAM (Figure 1b,c). PrP^C^-levels in spleen showed a considerable variation with a peak of PrP^C^ expression at day 60 after sham inoculation (Figure 1b,c, p = 0.04).

### EAM does not affect incubation time until onset of terminal prion disease

Having established a reliable EAM-model, we analyzed if EAM changed incubation time until onset of terminal prion disease. For this, we induced EAM and inoculated mice at day 21 after immunization with RML prions or sham. At identical time points, non-immunized SJL/J mice were treated equally as control. There was no difference in incubation times until onset of terminal prion disease between myosin-treated and non-treated mice (Figure 3a). EAM mice came down with disease after 182 +/− 8 days and non-immunized mice after 177 +/− 3 days.

After a proteinase K digestion of tissue homogenates we could detect presence of variable amounts of PrP^Sc^ by Western blot analysis in muscle, brain and spleen with no obvious differences in PrP^Sc^ loads and PrP^Sc^ glycotypes between EAM and controls (Figure 3b, Additional file 2: Figure S2).

In terminally sick mice, where the peak of active myositis has passed, morphological changes such as necrotic muscle fibers or fibrosis, typical for post-myositic muscles, were observed in EAM mice only (Figure 3c). In brain, spongiform changes and astrogliosis, typical for terminal prion disease were observed in both cohorts at comparable extent (Figure 3c).

### EAM leads to transient PrP^Sc^ accumulation in muscle in subclinical prion disease

In order to analyze if EAM leads to changed kinetics of PrP^Sc^ accumulation in muscle, we investigated PrP^Sc^ loads by NaPTA-precipitation and Western blot analysis after proteinase K digestion to detect PrP^Sc^ at 35, 60 and 90 days post prion challenge (Figure 4a). At 35 as well as at 60 days after prion inoculation, we detected prominent PrP^Sc^ accumulation only in EAM mice, whereas we could not detect significant levels of PrP^Sc^ in control mice. Interestingly, at 90 days after prion inoculation, none of the muscle samples showed any PrP^Sc^ accumulation (Figure 4a), whereas at terminal disease, PrP^Sc^ accumulation was equally strong in EAM and control mice (Figure 3b). In brain and spleen PrP^Sc^ accumulation was not influenced by EAM with high PrP^Sc^ contents in spleen at all examined time points and rising PrP^Sc^ contents in brain detectable from day 90 onwards (Figure 4a).

Since autophagy has been shown to clear PrP^Sc^ effectively, we investigated if increased autophagy may help to clear PrP^Sc^ in muscle. For this, we determined LC3 levels in C57/Bl6 mice at day 0 in order to monitor if autophagy is per se activated in muscle tissue versus brain and spleen. As shown in Figure 4b, there was a high up regulation of LC3 II, a marker for autophagy, in muscle when compared to brain and spleen.

Histologically, signs of myositis could be observed in muscle of EAM mice, but not in control animals at days 35 and 60. At day 90 only EAM mice showed necrotic muscle fibers and fibrosis, both of which are routinely observed as late signs of myositis (Figure 4c). In brain no obvious pathological changes could be detected at preclinical stages (Figure 4c).

### Dissociation of PrP^Sc^ loads and prion infectivity titers in muscle

PrP^Sc^ is thought to constitute an essential component of prion infectivity. Nevertheless, presence of prion infectivity does not strictly correlate with PK-resistant PrP^Sc^ load and protease-sensitive PrP^Sc^ species harbor significant amounts of prion infectivity [38]. Thus we assessed prion titers of muscle and spleen by bioassay (Figure 4c). Titers of infectious prions in muscle were under the detection limit of the assay at early time points demonstrating partial dissociation of PrP^Sc^ loads and prion titer. At day 90, prion titers were around LD50 for both cohorts with one EAM mouse (at day 129) and two control mice (at days 123 and 160) showing signs of prion disease. However, at terminal disease stages high infectivity titers (4.1 log LD50/ml of tissue homogenate for EAM, 4.3 log LD50/ml of tissue homogenate for control) could be measured in muscle. As expected, in spleen high infectivity titers (3.8, 4.6, 4.8 and 5.2 log LD50/ml tissue homogenate for EAM, and 4.5, 4.8, 4.7 and 4.9 log LD50/ml tissue homogenate for controls) at days 30, 60, 90, and at terminal disease could be detected irrespective of the presence of EAM.

### PrP^Sc^ accumulation in muscle at early time points requires a PrP^C^ expressing lymphoreticular system

Next, we set out to examine which cell type in muscle tissue is responsible for accumulation of PrP^Sc^. Since we could show that elevated PrP^C^ levels in muscle occur during peak of myositis (Figure 1c), cells of the hematopoietic compartment were likely candidates. In order to further investigate the role of PrP^C^ in the LRS we generated bone marrow chimeras with a PrP^C^-expressing and PrP^C^-deficient LRS. Since *Prnp*^*0*/*0*^ mice are kept on a C57Bl/6 background, and the majority of EAM models use a SJL/J background [33], we first had to establish a protocol for the induction of EAM in C57Bl/6 mice, thus allowing transplantation of syngenic bone marrow.

EAM in C57Bl/6 mice was confirmed by FACS analysis of muscle at 21 days post induction showing increased presence of T-cells (9.1% CD3-positive cells for EAM; 4.4% CD3-positive cells for controls, see Table 3). Upon histological analysis, we could observe T-cell dominant perimysial infiltrates in EAM in C57Bl/6 mice (Figure 2). Interestingly, we could not observe MFG-E8-positive FDCs and only a few B-cells.

**Table 3:** **FACS analysis of muscle homogenates from C57Bl**/**6 mice at day 21 after myosin infection or without myositis as a control**

Infiltrating cells (in %)	Control	21 days
T-cells	4.4	9.1
B-cells	7.0	0.7
Other lymphocytes	7.1	2.1
Neutrophile granulocytes	17.8	37.1

Values are the percentages of every immune cell regarding to the total amount of infiltrating cells.

To study PrP^Sc^ accumulation, we lethally irradiated C57Bl/6 mice and reconstituted their bone marrow with bone marrow of either *Prnp*^*0*/*0*^ or C57Bl/6 mice as a control. The reconstitution efficiency was assessed by FACS analysis of blood samples taken three weeks after lethal irradiation (Additional file 3: Figure S3). Afterwards, we induced EAM in 50% of the mice of both groups (*Prnp*^*0*/*0*^ → C57Bl/6; C57Bl/6 → C57Bl/6) and challenged them with either RML prions or mock at the peak of myositis. Accumulation of PrP^Sc^ and infectious prions was investigated by serially sacrificing mice at defined time points (day 35 and 90) and detection of muscular PrP^Sc^ loads by NaPTA-precipitation and Western blots. Only in mice with a PrP^C^-expressing hematopoietic compartment and EAM, we were able to observe significant accumulation of PrP^Sc^ in muscle at day 35 (Figure 5a). At day 90, no significant accumulation of PrP^Sc^ could be detected in either group. In spleen, PrP^Sc^ could be detected at day 35 and day 90 irrespective of the PrP^C^ status of the hematopoietic compartment (Figure 5a).

In brain, as expected at this preclinical state of the disease, no spongiform changes or astrogliosis was seen (Figure 5b). In muscle, signs of myositis could be observed in both chimeric cohorts of mice after EAM but not in control mice at day 35 and 90 (Figure 5b). Since myositis is very mild in C57/Bl6 mouse lines compared to SJL/J mice, immune cells infiltrate singular muscle fibres (first, third and fourth row of pictures) or only the interstitial space (third row of pictures). Most infiltrating cells are T-cells as shown with the immunohistochemical staining of marker CD3. PrP^C^ loads are not detectable in muscle tissue by standard PrP^C^ staining, since expression levels are under the detection limit of this method.

To study the correlation between PrP^Sc^ loads and titers of infectious prions, we assayed prion titers of muscle and spleen by bioassay. As shown in Figure 5c, no infectivity was detectable at day 35 whereas at day 90 individual mice came down with prion disease with an attack rate below 50%, indicating that prion titers in these tissues were at the detection limit of the assay. As expected, in spleen high infectivity titers could be detected at all given time points irrespective of PrP^C^ expression in the LRS and myositis.

## Discussion

Although, the central nervous system is the principal site of prion accumulation and replication, and the only site where prion-related tissue damage is seen, PrP^Sc^ and prion infectivity can be found in peripheral compartments such as spleen and muscle. Research focusing on mechanisms of prion accumulation in the periphery has yielded important insights into the temporal kinetics and prerequisites of prion accumulation [39]. Presence of PrP^Sc^ and prion infectivity in muscle has been highlighted by a number of reports describing its presence in a wide range of instances such as sporadic and variant CJD, BSE, CWD and Scrapie [12, 13, 15, 29, 40, 41]. PrP^Sc^ in muscle can be found preclinically in prion-infected rodents and primates [31, 42]. Both, nerve fibres and myocytes have been shown to harbour PrP^Sc^ within muscle at terminal disease stages [13, 15, 42], yet in one patient with prion disease and myositis, PrP^Sc^ accumulation in muscle was surprisingly high [29]. In summary, the pathophysiological events underlying accumulation of PrP^Sc^ and prion infectivity in muscle specifically also in myocytes is poorly understood and it is likely that events predisposing to prion colonization of muscle at terminal disease differ from those at early subclinical time points. Detailed knowledge of the mechanisms controlling prion colonization of muscle may help to explain the role of muscle in neuroinvasion of prions from the periphery to the brain [43, 44]. Here, we studied the temporal kinetics of PrP^Sc^ accumulation and prion titers in two murine EAM models and generated bone marrow chimeras to investigate the role of PrP^C^ expression in the lymphoreticular system in myositis.

As expected, in our EAM models, the peak of myositis was reached 21 days following immunization [33]. At this time point, we could observe significantly elevated levels of PrP^C^ in muscle by Western blot yet not by immunohistochemical methods. Increased expression of PrP^C^ has been linked to a number of diseases of the muscle such as inclusion body myositis, dermato-, and polymyositis [25, 26]. Our data imply that this increase is transient coinciding with inflammation. Although, presence of myositis did not influence disease kinetics with regard to incubation times until terminal prion disease, we could observe that presence of PrP^Sc^ at early subclinical disease is augmented in EAM. Differences were most drastic at days 35 and 60 following prion challenge. Here, we could only detect very low amounts of PrP^Sc^ by NaPTA-enhanced Western blotting in controls, whereas mice with EAM showed high (at day 35) and moderate (day 60) PrP^Sc^ loads in muscle. Surprisingly, at a time point where the peak of myositis has passed (day 90), PrP^Sc^ was completely cleared from muscle. Although, we did not investigate the mechanisms of PrP^Sc^ clearance in detail, autophagy may contribute to this as cell culture data [45] and our *in vivo* data show higher rate of basal autophagy in muscle.

Preclinical PrP^Sc^ accumulation in muscle has been demonstrated in a number of instances, yet we are not aware of any study showing clearance of PrP^Sc^ from this compartment after prion colonization has been established [31, 42, 46].

We could observe an uncoupling of PrP^Sc^ and prion infectivity in muscle at day 35 and 60, when significant levels of PrP^Sc^ in EAM muscle occur in the absence of prion infectivity. Similar findings have recently been reported in the brain [47] and spleen [38]. Thus, our data expand the range of tissues where PrP^Sc^ and prion infectivity are not congruent and support the concept that non-PrP^Sc^ species considerably contribute to prion infectivity [47, 48].

The fact that EAM does not influence incubation time until onset of terminal prion disease is not surprising. Even if absence of PrP^Sc^ accumulation or prion infectivity in lymphoid tissue dramatically slows down development of prion disease, augmentation of prion replication even in ectopic sites does not lead to shortening of incubation time until onset of terminal prion disease [49, 50]. At terminal prion disease, PrP^Sc^ and prion infectivity could be observed irrespective of the presence of EAM. This is in line with a wealth of papers detecting PrP^Sc^ in muscle [12, 13, 29]. The fact that PrP^Sc^ is cleared from muscle and reappears at terminal disease fits to a concept where preclinical PrP^Sc^ results from lymphatic spread whereas PrP^Sc^ in diseased animals is due to centrifugal spread from the central nervous system [17, 43].

It has been suggested that glycosylation patterns of PrP^Sc^ differ between muscle and CNS [13]. We could not observe such differences with muscular and brain-derived PrP^Sc^ showing similar glycosylation patterns.

In the LRS, accumulation of PrP^Sc^ and prion infectivity has been associated with FDC networks [20, 51]. Since myositis leads to diffuse and not follicular lymphoid infiltrates, we did not find FDC networks, but rather single MFG-E8-positive cells in our EAM muscles. It is questionable if these represent *bona fide* FDCs or merely MFG-E8-positive macrophages. In either case, accumulation of PrP^Sc^ in muscle of EAM mice at early time points occurs in the absence of FDC-networks.

Our experiments with bone marrow chimeras generating mice with a PrP^C^-deficient LRS indicate that PrP^C^ expression on cells of the LRS is decisive for early, EAM-dependent accumulation of PrP^Sc^ in muscle. This sets early inflammation-modulated PrP^Sc^ accumulation in muscle apart from early PrP^Sc^ accumulation in spleen where PrP^Sc^ accumulates irrespective of the PrP^C^ status of the LRS [35]. In the spleen this property has been attributed to FDCs, which are of non-hematopoietic origin and radio-resistant [35]. Thus, it is plausible that in muscle, lack of FDC-networks creates a situation, where a PrP^C^-expressing LRS is needed for PrP^Sc^ accumulation.

These data imply that myocytes are unlikely candidates for accumulation of PrP^Sc^ in EAM at early time points. A hypothesis that would accommodate current experimental data, would conceive PrP^Sc^ accumulation in muscle as a heterogeneous, disease stage-dependent event. In early disease stages, PrP^Sc^ accumulation is driven by cells of hematopoietic origin, whereas at late disease stages, non-hematopoietic cells such as nerve fibres or myocytes represent the main sites for PrP^Sc^ accumulation [13, 15, 17, 18, 31, 52]. There have been major concerns regarding biosaftey aspects of muscle biopsy in subclinically prion-diseased patients [12, 29]. Although our data indicate that enhanced PrP^Sc^-loads in muscle may not be reflected by elevated prion titers it is not possible to interpolate our data from mouse experiments to the human situation and more research efforts are needed to assess biosaftey aspects of muscle biopsy in demented patients.

## Conclusions

Our data show that: (i) myositis positively influences PrP^Sc^ accumulation in homogenized muscle tissue at preclinical time points and that a PrP^C^-expressing LRS is a prerequisite for this, (ii) PrP^Sc^ and prion infectivity are uncoupled in muscle with detectable PrP^Sc^ in the absence of prion infectivity at preclinical time points, (iii) muscle, unlike the LRS, has an intrinsically high ability to clear PrP^Sc^ once myositis has ceased, possibly involving autophagy.

## Electronic supplementary material

Additional file 1: Figure S1: Hanging wire test from RML infected versus untreated mice with and without myositis. (PDF 108 KB)

Additional file 2: Figure S2: Analysis of the glycosylation type of PrP^Sc^ in muscle, brain and spleen. (PDF 65 KB)

Additional file 3: Figure S3: Examples of FACS analysis for the expression of PrP^C^ in blood of C57Bl/6, Prnp^0/0^, tga20 mice and bone marrow chimeras. (PDF 115 KB)
