# Supplementary material for: Myositis facilitates preclinical accumulation of pathological prion protein in muscle
Source: Acta Neuropathol Commun. 2013 Dec 3;1(1):78. doi: 10.1186/2051-5960-1-78 (PMC4046662; doi:10.1186/2051-5960-1-78)
Supplement: Supplementary file 1 — Additional file 1: Figure S1: Hanging wire test from RML infected versus untreated mice with and without myositis. (PDF 108 KB) [file 40478_2013_74_MOESM1_ESM.pdf]

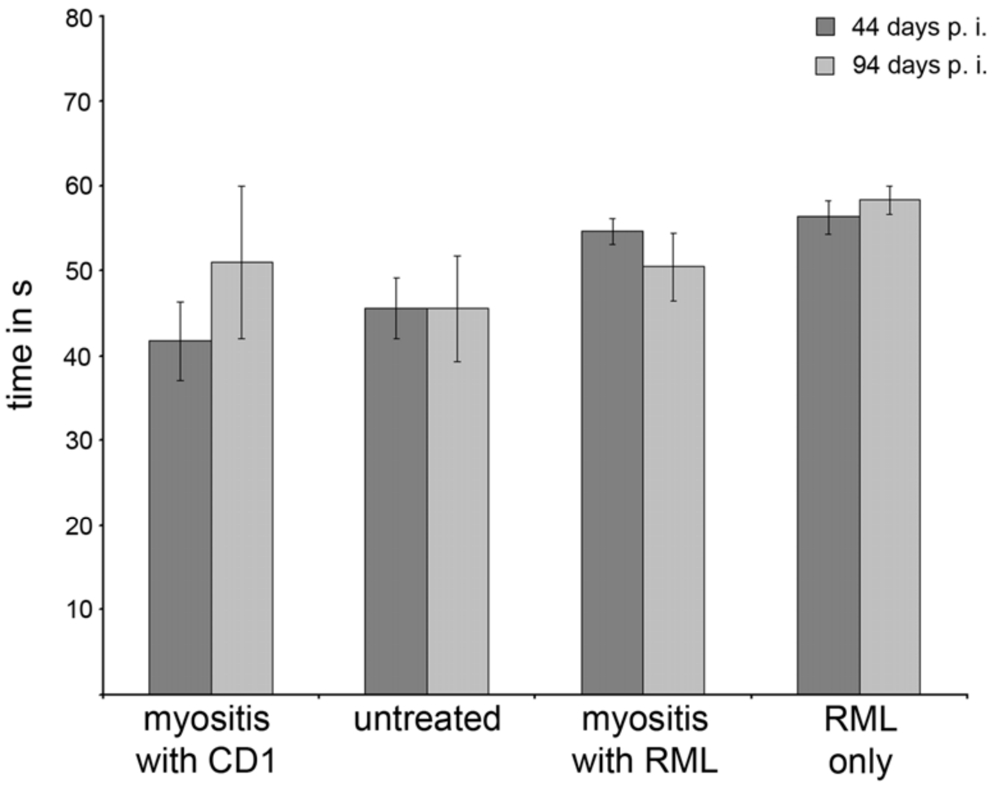

Additional file 1: Figure S1

Hanging wire test from RML infected versus untreated mice with and without myositis.

A hanging wire test was used to measure muscle strength in all analyzed groups o f mice to investigate if myositis affects mu scle strength. There were no differences in muscle strength in all groups of mice at 44 and at 94 days after inoculation with RML.
