# Supplementary material for: Myositis facilitates preclinical accumulation of pathological prion protein in muscle
Source: Acta Neuropathol Commun. 2013 Dec 3;1(1):78. doi: 10.1186/2051-5960-1-78 (PMC4046662; doi:10.1186/2051-5960-1-78)
Supplement: Supplementary file 2 — Additional file 2: Figure S2: Analysis of the glycosylation type of PrPSc in muscle, brain and spleen. (PDF 65 KB) [file 40478_2013_74_MOESM2_ESM.pdf]

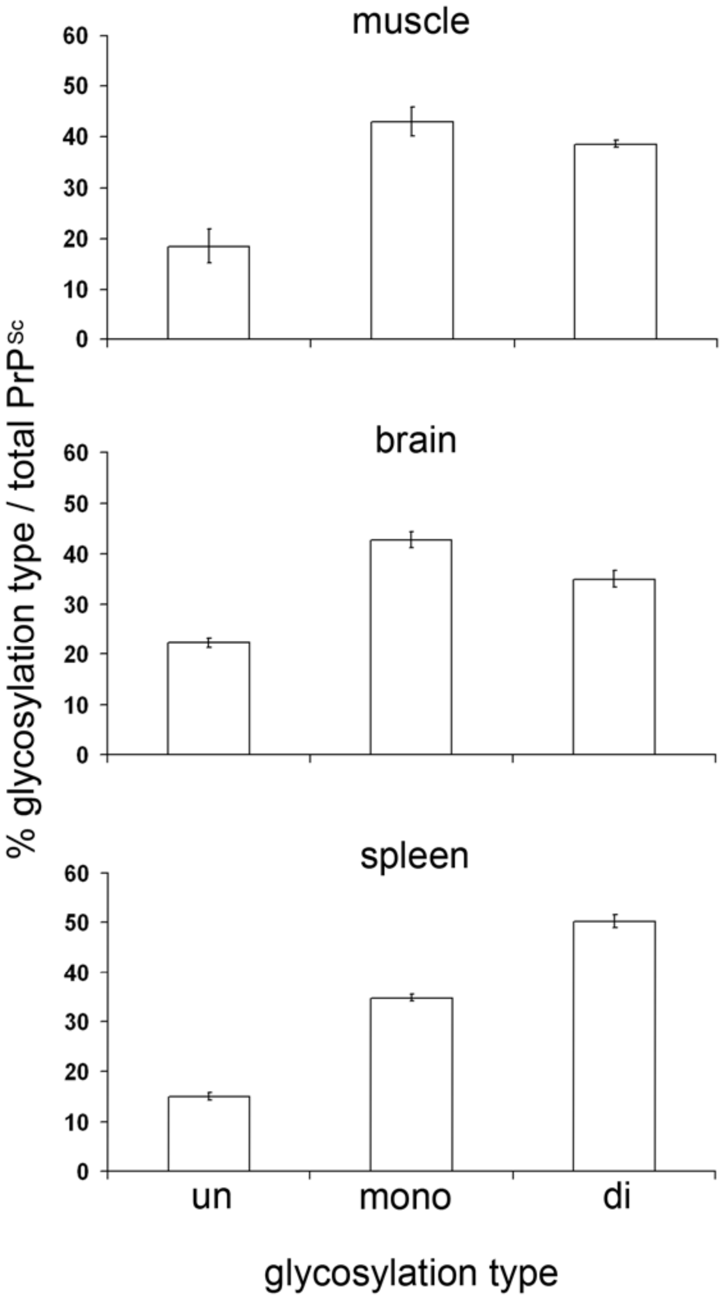


**Additional file 2: Figure S2**

*Analysis of the glycosylation type of PrP$^{Sc}$ in muscle, brain and spleen*.

The relative percentages of un- mono- and di-glycosylated PrP$^{Sc}$ were measured in NaPTA blots for muscle and brain as well as in Western blots for spleen. In muscle and brain the mono-glycosylated form of PrP$^{Sc}$ is dominant, whereas in spleen the di-glycosylated form of PrP$^{Sc}$ is more pronounced.
