# Supplementary material for: Myositis facilitates preclinical accumulation of pathological prion protein in muscle
Source: Acta Neuropathol Commun. 2013 Dec 3;1(1):78. doi: 10.1186/2051-5960-1-78 (PMC4046662; doi:10.1186/2051-5960-1-78)
Supplement: Supplementary file 3 — Additional file 3: Figure S3: Examples of FACS analysis for the expression of PrPC in blood of C57Bl/6, Prnp0/0, tga20 mice and bone marrow chimeras. (PDF 115 KB) [file 40478_2013_74_MOESM3_ESM.pdf]

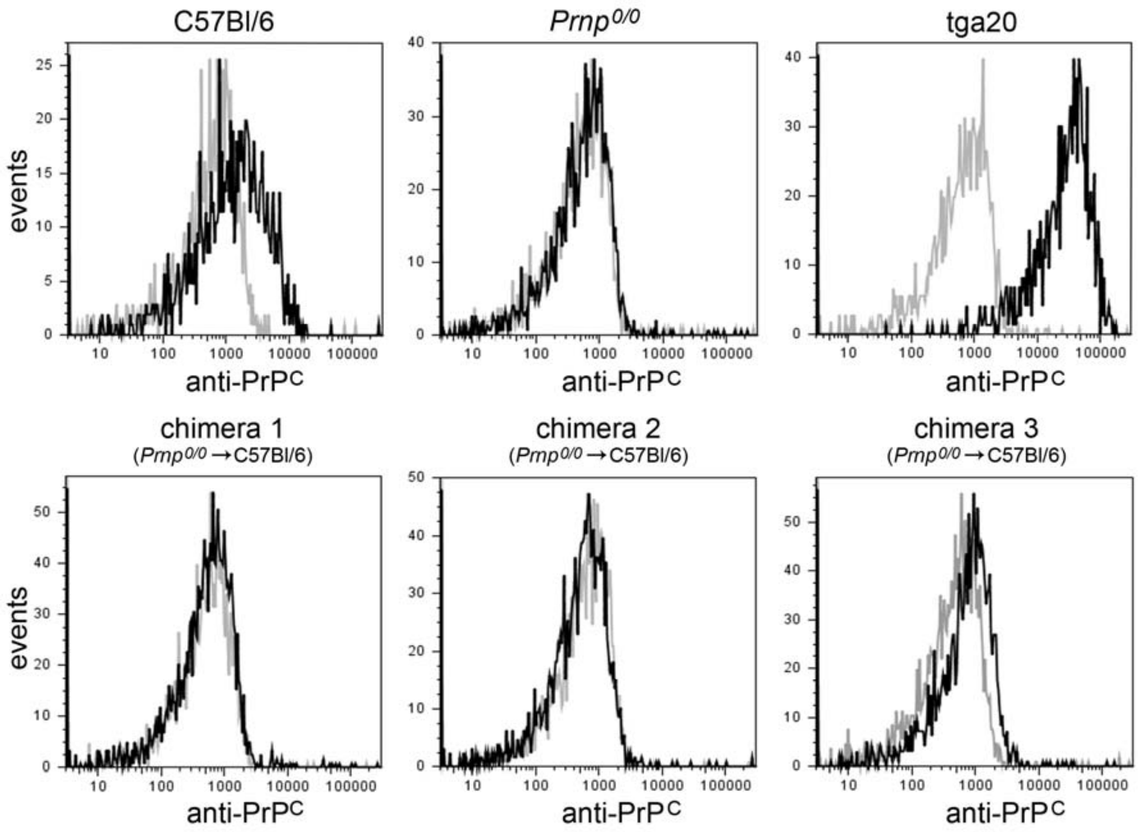

Additional file 3: Figure S3

Examples of FACS analysis for the expression of PrP^C in blood of C57Bl/6, $Prnp^{0/0}$, tga20 mice and bone marrow chimeras.

Bone marrow chimeras engrafted with bone marrow of *$Prnp^{0/0}$* mice show no significant shifts of their PrP fluorescence intensity peak indicating that at least 95 % of their LRS is PrP^C-deficient bone marrow. As controls C57Bl/6 mice show a shift of 5000 to 20000 and tga20 mice show a shift from 5000 to 200000 in their PrP fluorescence intensity peaks when compared to the iso-type control (grey line in all graphs).
